# The Transplantation of the Mucous Membrane

**Published:** 1888-08

**Authors:** 


THE TRANSPLANTATION OF THE MUCOUS MEMBRANE.—At the recent Congress of German Surgeons, Wolfler, of Gratz, spoke of the value of transplantations of mucous membrane. He said that where cicatrices are produced in cylindrical organs of the body, which are lined with mucous membrane, an operation could only be useful if it were possible, after excision of the scar, to unite the mucosa. But that is impossible in many cases. Therefore he had employed the method of Thiersch (the transplantation of portions of epidermis) with great success. After having excised the thickened and indurated tissue from impermeable urethral strictures, he transplanted mucous membrane from a prolapsed uterus. The operation was completely successful. In the same manner he transplanted mucus membrane from a prolapsed rectum on to the conjunctiva in a case of blepharoplasty. He succeeded even in transplanting mucous membrane from frogs, pigeons, and rabbits with good results.—*British Medical Journal*, April 14, 1888.

